# RG108 increases NANOG and OCT4 in bone marrow-derived mesenchymal cells through global changes in DNA modifications and epigenetic activation

**DOI:** 10.1371/journal.pone.0207873

**Published:** 2018-12-03

**Authors:** Rahyza I. F. Assis, Malgorzata Wiench, Karina G. Silvério, Rodrigo A. da Silva, Geórgia da Silva Feltran, Enilson A. Sallum, Marcio Z. Casati, Francisco H. Nociti, Denise C. Andia

**Affiliations:** 1 Department of Prosthodontics and Periodontics, Piracicaba Dental School, University of Campinas, Piracicaba, São Paulo, Brazil; 2 School of Dentistry, School of Cancer Sciences, University of Birmingham, Birmingham, United Kingdom; 3 Department of Chemistry and Biochemistry, Biosciences Institute, São Paulo State University, Botucatu,São Paulo, Brazil; 4 Division of Epigenetics, School of Dentistry, Health Science Institute, Paulista University, São Paulo, Brazil; Barts and The London School of Medicine and Dentistry Blizard Institute, UNITED KINGDOM

## Abstract

Human bone marrow-derived mesenchymal stem cells (hBMSCs) are important for tissue regeneration but their epigenetic regulation is not well understood. Here we investigate the ability of a non-nucleoside DNA methylation inhibitor, RG108 to induce epigenetic changes at both global and gene-specific levels in order to enhance mesenchymal cell markers, in hBMSCs. hBMSCs were treated with complete culture medium, 50 μM RG108 and DMSO for three days and subjected to viability and apoptosis assays, global and gene-specific methylation/hydroxymethylation, transcript levels’ analysis of epigenetic machinery enzymes and multipotency markers, protein activities of DNMTs and TETs, immunofluorescence staining and western blot analysis for NANOG and OCT4 and flow cytometry for CD105. The RG108, when used at 50 μM, did not affect the viability, apoptosis and proliferation rates of hBMSCs or hydroxymethylation global levels while leading to 75% decrease in DNMTs activity and 42% loss of global DNA methylation levels. In addition, *DNMT1* was significantly downregulated while *TET1* was upregulated, potentially contributing to the substantial loss of methylation observed. Most importantly, the mesenchymal cell markers CD105, NANOG and OCT4 were upregulated being NANOG and OCT4 epigenetically modulated by RG108, at their gene promoters. We propose that RG108 could be used for epigenetic modulation, promoting epigenetic activation of NANOG and OCT4, without affecting the viability of hBMSCs. DMSO can be considered a modulator of epigenetic machinery enzymes, although with milder effect compared to RG108.

## Introduction

The promising results of clinical tests using human mesenchymal stem cells (hMSCs) make them a relevant source for cellular therapy applications in a wide range of diseases and in regenerative medicine. However, the epigenetic regulation underlying hMSCs is not well understood. Pluripotency genes, such as POU5F1-POU-class-5-homeobox-1 (*OCT4*) and NANOG-Homeobox (*NANOG*), are expressed in hMSCs [1,2] and constitute the core regulatory network that suppresses differentiation-associated genes, thereby maintaining the pluripotency of cells [3]. *OCT4*/*NANOG* genes were demonstrated to be involved in inhibiting spontaneous differentiation in MSCs, via upregulating DNA methyltransferase 1 (DNMT1) [4]. In addition, Ten eleven translocation 1 (TET1), an enzyme involved in active DNA demethylation, is a NANOG protein partner and both proteins are associated with maintenance of pluripotency and lineage commitment in embryonic stem cells [5]. TET1 is also recruited by NANOG to enhance the expression of OCT4 [5]. Although recent investigations have identified fundamental mechanisms involved in the maintenance of the undifferentiated cell state, few studies have been focused on hMSCs.

Epigenetics refers to a change in a gene that is passed on through cell division but does not involve DNA sequence change [6]. Chemical changes in the DNA molecule and histone proteins may act by modifying the chromatin architecture and changing the accessibility of transcription factors to gene regulatory elements. Therefore, these modifications are essential for either maintaining cells in an undifferentiated state or directing them towards cell fate specification. DNA methylation (5mC) and hydroxymethylation (5hmC) represent the most studied epigenetic modifications within the DNA molecule itself. DNMT enzymes are implicated in the DNA methylation mechanism and there is evidence that DNMT1 acts as a maintenance methyltransferase, operating at replication forks [7]. DNMT3A and DNMT3B enzymes are involved in the *de novo* acquisition of DNA methylation status, introducing cytosine methylation at previously unmethylated CpG sites [7,8]. On the other hand, the oxidation of methylcytosine to hydroxymethylcytosine has been shown to underlie the active DNA demethylation mechanism and TET enzymes are responsible for catalysing this reaction [9]. Generally, unmethylated DNA is associated with permissive and open chromatin architecture, whereas the presence of nucleosomes and condensed chromatin is related to methylated DNA at gene promoters. However, the specific roles of epigenetic machinery enzymes in hMSCs require investigation.

Since epigenetic control is closely related to gene regulation, epigenetic modulators have been used to affect gene expression patterns and cell fate. RG108 [2-(1,3-dioxoisoindolin-2-yl)-3-(1H-indol-3-yl) propanoic acid or N-Phthalyl-L-tryptofan] is a compound specifically designed to block the active site of DNMTs [10]. The confirmed ability to reactivate several tumour suppressor genes and the lack of cell toxicity [10,11] make RG108 a good candidate for epigenetic modulation therapies. Several investigations have used RG108 for studying mechanisms involved in human disorders as well as in physiological conditions [12–18]. However, only two studies have tested this compound in hMSCs. In 2015, Oh et al. reported that RG108 could provide beneficial efficacy in treating ageing-related diseases by restoring the altered methylation pattern [19]. In the follow up study, they showed that senescence phenotype brought by excessive DNMTs’ expression found in amyotrophic lateral sclerosis could be controlled by RG108 [20]. The authors suggested that RG108 may provide a better efficacy of hMSCs in stem cells therapy, since the cell function was restored with an improvement in their stem cell potency, cell migration, oxidative stress protection and senescence.

Although RG108 was specifically designed to target DNMT enzymes, its effects on the epigenetic machinery itself as well as genome-wide effects are still not fully recognised. In addition, epigenetic regulation in hMSCs is also not completely understood. Therefore, in this study we investigate the ability of RG108 to trigger global changes in DNA methylation and hydroxymethylation in hMSCs, in addition to gene-specific effects on epigenetic machinery and NANOG and OCT4. The study aims at better understanding the epigenetic regulation in hMSCs and explores the possibility of using RG108 in hBMSCs-based cell therapies.

## Materials and methods

### Cell culture and differentiation

Cryopreserved human bone marrow-derived mesenchymal stem cells (hBMSCs) were purchased from Lonza (Walkersville, MD, USA) at passage 2 (P2) and tested by Lonza for the presence of viral nucleic acid from HIV, hepatitis B virus, and hepatitis C virus. Initially, cells were seeded at 5,000–6,000 per cm^2^ and grown in complete culture medium composed of Dulbecco’s modified Eagle high glucose medium (DMEM) with L-glutamine, supplemented with 10% fetal bovine serum (FBS), penicillin (100 U/ml) and streptomycin (100 mg/mL) (Gibco, Carlsbad, CA, USA). Cells were maintained in a humidified incubator at 37°C and 5% CO_2_ atmosphere.

Only cells at passages P5—P7 were used and cell synchronization was performed by serum starvation for 24 h, after each initial plating for each experiment. The age of hBMSCs donors was between 20 and 22 years.

To evaluate the cell phenotype, the expression of cell surface markers such as CD105/APC, CD166/PE, CD34/FITC and CD45/PerCP (BD Biosciences, San Diego, CA, USA) were analysed by flow cytometry using a FACSCalibur flow cytometer connected to the CellQuest program (BD Biosciences, USA).

In order to confirm multipotentiality, the cells were induced towards osteogenic and adipogenic differentiation as previously described [21]. For *in vitro* osteogenic differentiation, cells were cultivated in osteogenic induction medium (Lonza, Walkersville, MD, USA) for 21 days. In sequence, the Alizarin Red staining (AR) assay was performed. The quantification was measured in a spectrophotometric apparatus, at 562 nm, by comparing the samples to a standard curve using a micro-plate reader (VersaMax, Molecular Devices, Sunnyvale, CA, USA). In addition, gene expression analysis of *RUNX2*, an osteogenic marker, was performed. For *in vitro* adipogenic differentiation, hBMSCs were cultured in adipogenic induction medium in according to the manufacturer’s recommendation, using the induction and maintenance adipogenic medium from LONZA (Walkersville, MD, USA), for 25 days. Afterwards, the cells were fixed with 10% formalin and stained with oil red O (Merck Millipore, Darmstadt, Alemanha). In addition, gene expression analysis of adipogenic gene marker *PPARgamma-2* was performed.

### RG108 preparation

RG108 was purchased from Sigma (St. Louis, MO, USA) and dissolved in DMSO (Sigma-Aldrich, St. Louis, MO, USA) to make 200 mM stock solution [12] and stored at -20°C until further use. For control purposes, hBMSCs were also cultivated in standard medium with DMSO (vehicle control).

### Groups

After cell and drug characterization phase, experiments were carried out for 3 days and three experimental groups were defined as follows:

- DMEM: hBMSCs were cultivated in complete culture medium, supplemented with 10% FBS, penicillin and streptomycin.
- DMSO: hBMSCs were cultivated in complete culture medium, supplemented with 10% FBS, penicillin, streptomycin and 0.025% DMSO.
- RG108: hBMSCs were cultivated in complete culture medium, supplemented with 10% FBS, penicillin, streptomycin and final concentration of 50 μM RG108.

### Viability assay

Three biological replicates were used for each experiment. hBMSCs were seeded at 0,5 x 10^4^ cells/well in a 96-well plate with standard medium and incubated for 24h. After the 24h incubation and subsequent cell adhesion, cell starvation was performed for another 24h and then the cell treatment was initiated. hBMSCs were cultivated for 7 days, with media change at day 3. Cells from the test group were cultivated with RG108 diluted in complete culture medium. In the first set of experiments, high concentrations of RG108, such as 400 μM, 800 μM, 1.6 mM, were tested in two hBMSCs populations, for 7 days. In the second set, we tested lower concentrations of RG108, such as 50 μM, 100 μM 200 μM, for 7 days in one hBMSCs population. In the third set, we confirmed the results observed for 50 μM RG108 at 7 days (where cell metabolic activity was the least affected) by performing three independent viability assays in one hBMSCs population with a 3 days treatment. The cell viability was determined by the metabolic activity using 3-(4,5-dimethylthiazol-2-yl)-2,5-diphenyltetrazolium bromide (MTT) assay (Sigma, St. Louis, MO, USA) according to the manufacturer’s instructions. Each of the biological replicates was plated six times (technical replicates) for absorbance reading at 540 nm using VersaMax micro-plate reader (Molecular Devices, Sunnyvale, CA, USA).

### Population doubling time assay

To determine the population doubling time (PDT), hBMSCs were seeded at 24 well plates at three different densities with standard medium with or without 50 μM RG108. The experiment was repeated three times. After adhesion, cells corresponding to the time 0 were fixed with 5% trichloroacetic acid, lysed with NaOH (0.5 mol/L) and cell quantification was determined with the spectrophotometric analysis at 260 nm. The remaining cells were fixed, lysed and quantified at 1, 2 and 3 days. The PDT was established by calculating the log natural l(n) for the initial concentrations. Then, from a l(n) linear regression (l(n)–dependent variable x time–independent variable) the angular linear coefficient was generated (μ). Calculations of PDT were done from the following equation g = ln2/μ, where ln2 is 0.30103.

### Apoptosis assay

Aiming to evaluate if RG108 at concentration of 50 μM and 100 μM could trigger cell death by apoptosis, 3 x 10^5^ cells were seeded in 100 mm dishes, cultivated in complete culture medium and treated according to experimental groups for 3 days and 7 days. Cells were harvested and freshly evaluated for apoptosis by flow cytometry using nucleic acid dye 7-Amino-Actinomycin D (7-AAD) and phospholipid-binding protein Annexin V (BD Pharmingen, San Jose, CA USA), as described by the manufacturer’s instruction. Cells were gated into four populations and counted using a FACSCalibur flow cytometer (BD Biosciences, San Jose, CA, USA) connected to the CellQuest software (BD Biosciences, USA). The non-apoptotic cells were sorted into the lower left quadrant.

### DNA, RNA and protein isolation

hBMSCs were seeded at 2.23 x 10^6^ cells in 150 mm dishes in complete culture medium and treated according to experimental groups. Two independent experiments were performed. After 3 days cells were scraped off in PBS and divided into three eppendorf tubes for DNA, RNA and protein extraction. DNA and protein tubes were stored at -80°C until further use. RNA tubes were quickly centrifuged in refrigerated centrifuge for pellet formation at 4°C after which 1 mL of TRIzol reagent (Gibco BRL, Life Technologies, Rockville, MD, USA) was added to the pellet and samples were stored at -80°C until further use.

Total RNA was isolated by TRIzol method, according to manufacturer’s recommendation, DNase-treated (Turbo DNA-free, Ambion Inc., Austin, TX, USA) and 1 μg was used for complementary DNA (cDNA) synthesis, using the First-Strand cDNA Synthesis Kit (Roche Diagnostic Co., Indianapolis, IN, USA). cDNA samples were stored at -20°C.

Total DNA was purified by extraction with phenol/chloroform/isoamyl alcohol and DNA samples were stored at -20°C. DNA and RNA concentrations were determined using spectrophotometer (Nanodrop 1000; Nanodrop Technologies LLC, Wilmington, NC, USA).

Total protein was extracted using EpiQuik Nuclear Extraction Kit I (Epigentek Group Inc., NY, USA) using 10^6^ cells as input and 20 μL as a final volume. Subsequently, protein nuclear extracts were quantified using Pierce BCA Assay (Thermo Scientific Inc., Brenner, Germany), according to manufacturer’s recommendation and stored at -80°C.

### Global methylation assay

Global DNA methylation level analysis was performed using Imprint Methylated DNA Quantification Kit (Sigma, St. Louis, MO, USA) as previously described [14] and according to manufacturer’s instructions. Each DNA sample was used in five technical replicates. Methylated control (50 ng/μL) was used for comparison of methylation levels (% of the methylated control) and the 450 nm absorbance was read in a VersaMax microplate reader (Molecular Devices, Sunnyvale, CA, USA).

### Global hydroxymethylation assay

For global hydroxymethylation levels, the Quest 5-hmC DNA Elisa kit (Zymo Research Corp., Irvine, CA, USA) was used and 100 ng DNA of each sample was plated five times for each group, as previously described [22] and according to manufacturer’s recommendation. The control DNA set containing a specified percentage of 5-hmC, supplied by the manufacturer, was used to determine the hydroxymethylation quantification and the 450 nm absorbance was read in VersaMax microplate reader (Molecular Devices, Sunnyvale, CA, USA).

### DNMTs activity assay

5 μg of protein nuclear extract input for each sample was used to analyse DNMTs activity in duplicate measurement with EpiQuik DNA Activity/Inhibition Assay Ultra Kit Colorimetric (Epigentek Group Inc., NY, USA), as previously described [23] and according to manufacturer’s recommendation. The 450 nm absorbance was read in VersaMax microplate reader (Molecular Devices, Sunnyvale, CA, USA).

### Demethylases activity assay

5 μg of protein nuclear extract input for each sample was used to measure the demethylases activity with Epigenase 5mC-Hydroxylase TET Activity/Inhibition Assay Kit (Epigentek Group Inc., NY, USA) in duplicate as previously described [23] and according to manufacturer’s recommendation. The 450 nm absorbance was read in VersaMax microplate reader (Molecular Devices, Sunnyvale, CA, USA).

### mRNA expression analysis

The *DNMT1*, *DNMT3A*, *DNMT3B*, *TET1*, *TET2*, *SOX2*, *NANOG* and *OCT4* mRNA expression analysis was conducted in a real-time polymerase chain reaction (RT-PCR) apparatus (LightCycler 480 Real Time PCR System; Roche Diagnostics GmbH, Mannheim, Germany) with a SYBR Green Kit (FastStart Essential DNA Green Master; Roche Diagnostic Co., Indianapolis, IN, USA). Results were expressed as relative amounts of the target gene using β-actin as inner reference gene. The details of the primers are presented in Table 1.

**Table 1:** Primer sequences used for RT-qPCR.

Gene	Primer	5’-3’ sequence	Reactions Conditions
*DNMT1*	Forward	GAGCCACAGATGCTGACAAA	95°C–10” 60°C– 10” 72°C– 10”
	Reverse	GACACAGGTGACCGTGCTTA	
*DNMT3A*	Forward	AAGGAGGAGCGGCCAGAG	95°C– 10” 58°C– 10” 72°C– 23”
	Reverse	GGATGGGGACTTGGAGATCA	
*DNMT3B*	Forward	GGGAGGTGTCCAGTCTGCTA	95°C– 10” 60°C– 10” 72°C– 18”
	Reverse	GGCTTTCTGAACGAGTCCTG	
*TET1*	Forwar	TCATGGGTGTCCAATTGCTA	95°C– 10” 61°C– 09” 72°C– 09”
	Reverse	GATGAGCACCACCATCACAG	
*TET2*	Forward	GGACATGATCCAGGAAGAGC	95°C– 10” 55°C– 10” 72°C– 18”
	Reverse	CCCRCAACATGGTTGGTTCT	
*TET3*	Forward	CCCACAAGGACCAGCATAAC	95°C– 10” 58°C– 10” 72°C– 23”
	Reverse	CCATCTTGTACAGGGGGAGA	
*NANOG*	Forward	AGGGCTGTCCTGAATAAGCA	95°C– 10” 56°C– 10” 72°C– 10”
	Reverse	GAGATGCCTCACACGGAGAC	
*OCT4*	Forward	CGCAAGCCCTCATTCAC	95°C– 10” 60°C– 06” 72°C– 06”
	Reverse	CATCACCTCCACCACCTG	
*SOX2*	Forward	GCAAACTTCCTGCAAAGCTC	95°C– 10” 57°C– 10” 72°C– 10”
	Reverse	GCAAACTTCCTGCAAAGCTC	
*ß-ACTIN*	Forward	CCAACCGCGAGAAGATGA	95°C– 10” 61°C—10” 72°C– 10”
	Reverse	CCAGAGGCGTACAGGGATAG	

### Quantitative PCR assay for gene-specific DNA methylation and hydroxymethylation

Genomic DNA was initially treated with T4-β-glucosyltransferase (T4-BGT) (New England Biolabs, Beverly, MA, USA), which adds glucose moiety to 5-hmC in order to distinguish amongst DNA methylation and hydroxymethylation. For each sample, three tubes containing 400 ng gDNA each were processed (1X NE buffer 4, 40 mM UDP glucose, 1U T4-BGT) in a final volume of 20 μL and incubated at 37°C for 1 hour, followed by 10 min at 65°C. Subsequently, samples were digested with methylation-independent *Msp*I or methylation-sensitive *Hpa*II restriction enzymes (New England Biolabs, Beverly, MA, USA) or H_2_O (control), to a final volume of 25 μL at 37°C for 1 hour. Tubes containing the *Hpa*II restriction enzyme were submitted to additional incubation for 10 min at 65°C, for enzyme inactivation. Subsequently, 40 ng of gDNA was subjected to 40 amplification cycles using 0.5 μM of gene-specific primers and gene-specific qPCR conditions (Table 2) in a final reaction volume of 10 μL. Amplicon locations for *DNMTs*, *TETs*, *OCT4* and *NANOG* were chosen at regulatory regions such as DNaseI hypersensitivity clusters sites, layered by histone modifications marks, CpG-enriched regions and transcription factors binding sites (https://genome.ucsc.edu). The primers were designed using Primer3 [24] and further analysed for secondary structures and annealing temperatures by the Beacon Designer, Free Edition (http://www.premierbiosoft.com/). Sequences and chromosome location were confirmed by the *in-silico* PCR (https://genome.ucsc.edu/). qPCRs were performed as described for mRNA expression. Each sample was run in three technical replicates. The comparative Ct method was used in analysis and samples were normalized by setting the control reaction (only treated with T4-BGT, without enzymes digestion) as a calibrator.

**Table 2:** Primer sequences for gene specific DNA methylation and hydroxymethylation qPCR.

Gene	Primer	5’-3’ sequence	Reactions Conditions	Product size (bp)	Restriction enzymes sites position and number of CCGG analysed	Number of CCGG excluded in the analysed region
*DNMT1*	Forward	TCGGGAGGCTTCAGCAGACG	95°C– 15” 63°C– 15” 72°C– 15”	201 bp	chr 19: 3 CCGG sites 10.194.943/10.195.008 10.195.079	0
	Reverse	TGCGTGTTCCCTGGGCAT				
*DNMT3A*	Forward	GTCCCCGCATCCAGCAC	95°C– 10” 57°C– 10” 72°C– 15”	200 bp	chr 2: 3 CCGG sites 25.335.394/25.342.503 25.342.541	0
	Reverse	CTGAGGCAGGCAGAGCG				
*DNMT3B*	Forward	TCAAATTTCCCTCGTCCCCG	95°C– 10” 60°C– 10” 72°C– 10”	129 bp	chr 20: 4 CCGG sites 32.762.284/32.762.322 32.762.343/32.762.348	8
	Reverse	GTGCCGACTCCCCTTGTAG				
*TET1*	Forward	GAGTTGGAAAGTTTGCCCGA	95°C– 10” 57°C– 10” 72°C– 10”	211 bp	chr 11: 4 CCGG sites 68.560.733/68.560.754 68.560.848/68.560.918	9
	Reverse	AGAGCCAAGGACGCAGAG				
*TET2*	Forward	CATCCCAACCTCCCACCTC	95°C– 10” 56°C– 10” 72°C– 15”	479 bp	chr 4: 10 CCGG sites 105.146.380/105.146.446 105.146.535/105.146.550 105.146.583/105.146.616 05.146.637/105.146.659 105.146.677/105.146.748	0
	Reverse	GGCGGACGTGACTTGCAT				
*TET3*	Forward	CTGTTTCCTAGCCGCATCAC	95°C– 10” 57°C– 10” 72°C– 15”	198 bp	chr 2: 1 CCGG site 74.002.302	18
	Reverse	CCACTCTCTAACCCCAGCAT				
*NANOG*	Forward	CGAGACATAGACTATCTGCCTGA	95°C– 8” 59°C– 8” 72°C– 8”	103 bp	chr 12: 1 CCGG site 7.787.998	0
	Reverse	TTCTTCTCAGACTACCATTCCG				
*OCT4*	Forward	GAAATCCGAAGCCAGGTGTC	95°C– 9” 63°C– 9” 72°C– 9”	195 bp	chr 6: 1 CCGG site 31.170.643	0
	Reverse	TCCTTCGCCTCAGTTTCTCC				

### Fluorescence microscopy

For microscopy analyses, the hBMSCs were cultured on glass coverslips and treated according to experimental groups described earlier. After 3 days, cells were washed with PBS, fixed in PBS-paraformaldehyde 4% for 1 h and permeabilized in PBS containing 0.2% Triton-X 100 and 1% BSA at 37°C for 1 h and stained with primary antibodies diluted according to the manufacturer’s instructions: mouse monoclonal OCT4 (ab-59545, Abcam; 1:100 dilution) and mouse monoclonal NANOG (1E6C4—sc-293121, Santa Cruz Biotechnology; 1:100 dilution) for 1 h at room temperature. After washing with PBS, the cells were stained with secondary antibody rabbit anti-mouse IgG H+L Alexa Fluor 488 (Invitrogen) for 1 h at room temperature. Cells were washed with PBS and coverslips were mounted on glass slides using Fluoroshield with DAPI (Sigma) and then viewed on Zeiss Axio Observer inverted microscope with Apotome.2 (Zeiss, Germany). The quantification of NANOG and OCT4 was performed as previously described [25].

### Western blotting analysis

The protein extracts were obtained and extracted in 500 μL Laemmli Buffer [SDS 4%, glycerol 20%, Tris-Cl (pH 6.8) 120 mM, bromophenol blue 0.02% (w/v) and DTT 0.1 M] and samples were stored at -20°C until further analyses. 15 mL protein (75 mg) was resolved by SDS-PAGE and blotted onto Immobilon FL PVDF membranes (Millipore, Bedford, MA, USA), blocked in Tris-buffered saline (TBS) with 0.05% Tween 20, albumin 2,5% (TBSTA) and incubated overnight at 4°C with NANOG (1E6C4—sc-293121, Santa Cruz Biotechnology) and OCT4 (ab-59545, Abcam) antibodies, followed by the appropriate horseradish peroxidase (HRP)-linked secondary antibody, at 1:5000 dilution, in TBSTA for 1 h. The immunoreactive bands were detected with enhanced chemiluminescence kit.

### Flow cytometry of CD105

To evaluate the RG108 and DMSO effects in the MSCs surface molecule CD105, its expression was analyzed by flow cytometry, after DMEM, RG108 and DMSO treatments for three days. Cells were detached using a cell dissociation enzyme-free buffer (TrypLE, Gibco, Carlsbad, CA, USA) to obtain single cell suspensions. Cells were suspended in blocking buffer for 40 minutes at 4°C. Approximately 3 x 10^5^ cells were incubated with mouse anti-human monoclonal antibody against CD105-allophycocianin (eBioscience, USA) or isotype-matched control for 40 min at 4°C. Following washing, cells were analyzed using a FACSCalibur flow cytometer (BD Biosciences, San Jose, CA, USA) connected to the CellQuest program (BD Biosciences, USA). The results were expressed as percentage of positive cells on 10,000 events.

### Statistical analysis

Data were initially examined for normality by Shapiro-Wilk test (or similar) and expressed as mean ± standard deviation (SD). After normal data distribution was confirmed, One-way analysis of variance (ANOVA α ≤ 0.05) followed by pairwise multiple-comparison test (Tukey) were used to identify the difference amongst groups; otherwise, the Kruskal-Wallis, followed by the Dunn's Multiple Comparison Test were employed (GraphPad Prism 7 –GraphPad Software Inc., San Diego, CA, USA).

## Results

### hBMSCs phenotyping and differentiation

**Fig 1:**
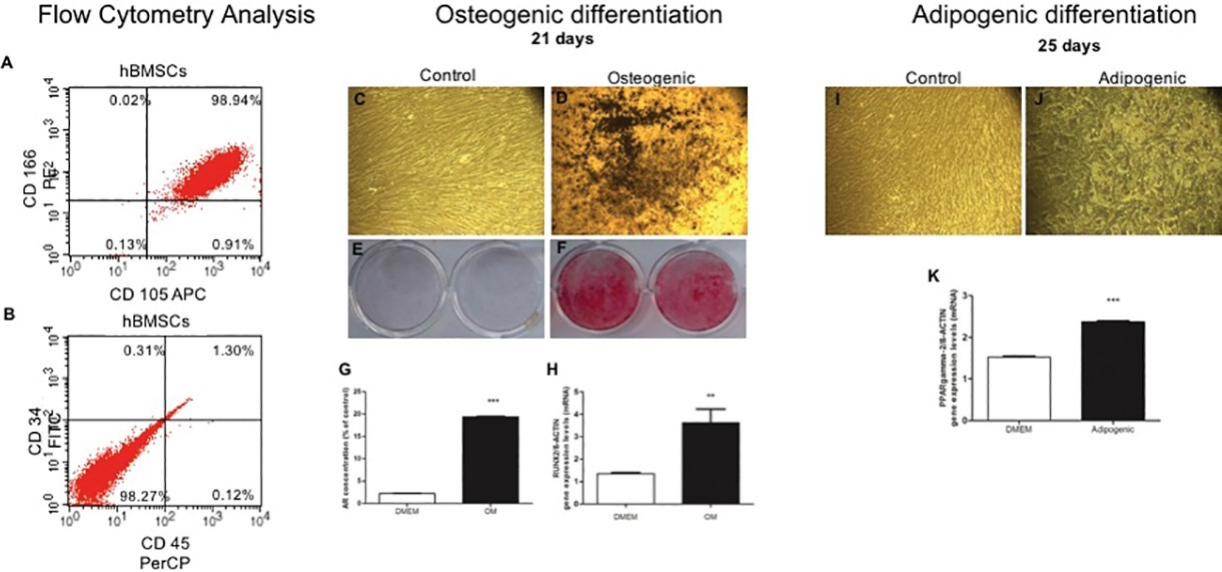
hBMSCs phenotyping and differentiation. Dot plot graphs illustrate the presence/absence of mesenchymal cell surface markers, performed by flow cytometry analysis on hBMSCs. (A) CD166 and CD105 positive cells are sorted into the upper right quadrant (UR) (98.94%) and (B) CD34 and CD45 negative cells are sorted into the lower left (LL) (98.27%). The results were obtained by flow cytometry performed in FACS Calibur flow cytometer (BD Biosciences, San Jose, CA, USA). (C) Representative photomicrograph of osteogenic differentiation with 10x magnification of control group (standard medium—DMEM 10%) and (D) osteogenic group (osteogenic induction medium). (E) Alizarin red staining after 21 days of osteogenic induction with standard culture medium—DMEM 10% and (F) osteogenic medium and (G) levels of alizarin red concentration in both groups; (H) RT-PCR analysis of RUNX2 gene expression. Representative photomicrograph of adipogenic differentiation (10x magnification) after 25 days of induction, (I) control group cultured in standard medium–(DMEM 10%) and (J) adipogenic induction medium; (K) RT-PCR analysis of gene expression of PPARgamma-2. For all graphics, * above the bars represent significant inter-group differences when compared to DMEM. ** and *** indicate, respectively, p ≤ 0.001 p ≤ 0.0001 by the test T student.

To confirm undifferentiated state of hBMSCs, flow cytometry analysis was performed, and the cells presented expression of CD105 and CD166 (98.94%). Additionally, hBMSCs showed lack expression of CD45 and CD34 (98.27%) (Fig 1). The osteogenic (Fig 1C–1H) and adipogenic (Fig 1I–1K) potential also was evaluated. The osteogenic induced-hBMSCs were able to differentiate into osteoblasts confirmed by the presence of mineral deposits after Alizarin red staining (p ≤ 0.0001) (Fig 1E–1G) at 21 days of osteogenic induction; in addition, the cells showed increase in the *RUNX2* transcript levels (Fig 1H), when compared to the control group (not induced) (p ≤ 0.001). The hBMSCs were able to differentiate into adipocyte, after 25 days of adipogenic induction (Fig 1J), presenting lipid vacuoles stained by oil Red O and increase in the transcript levels of *PPARgamma-2* (p ≤ 0.0001).

### Effect of RG108 on viability and apoptosis in human bone marrow-derived mesenchymal stem cells (hBMSCs)

**Fig 2:**
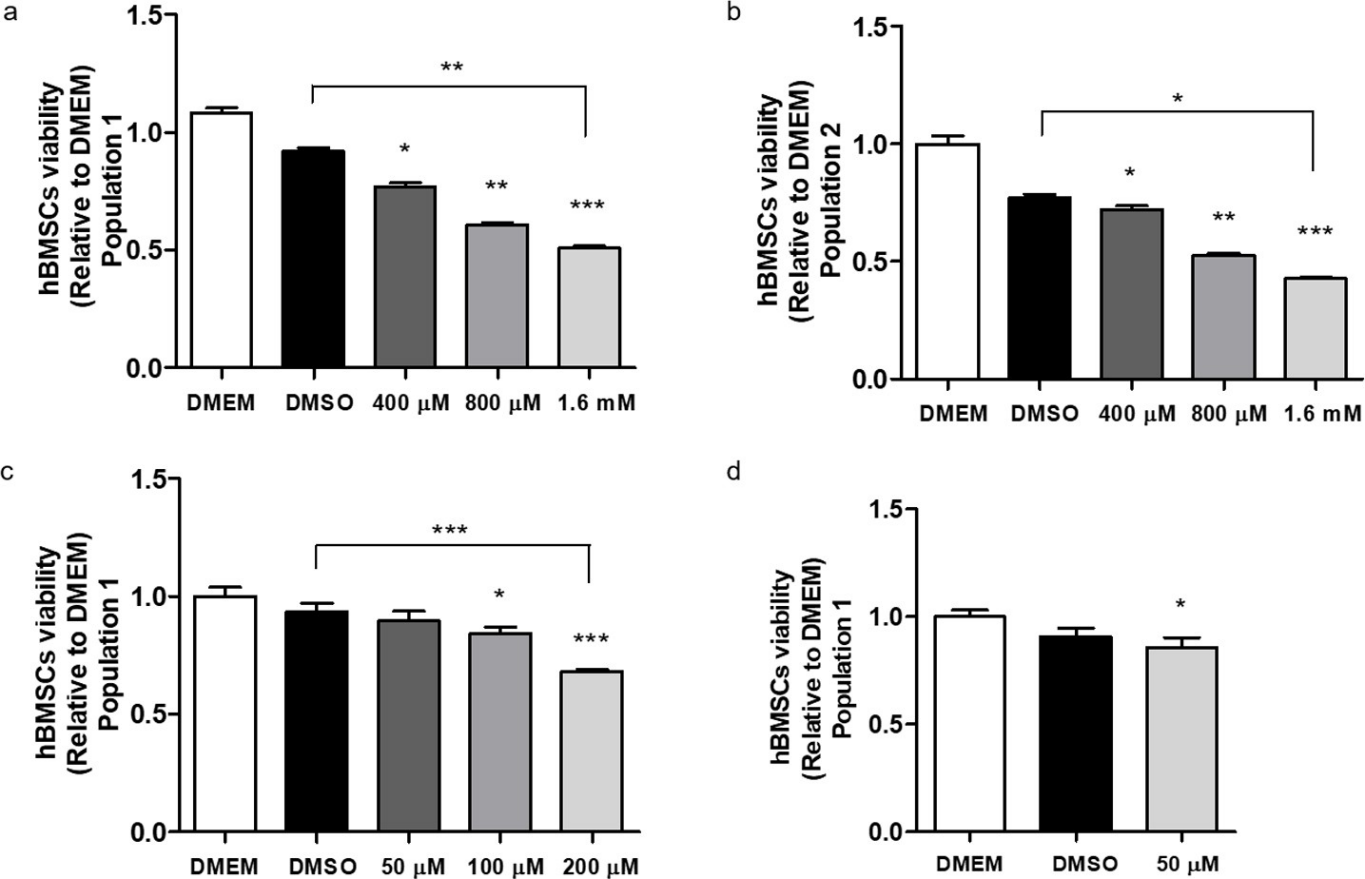
Effect of RG108 on hBMSCs cell viability. (A, B) 2 populations of hBMSCs were treated with 400 μM, 800 μM and 1.6 mM of RG108 for 7 days, showing significant reduction in viability comparing to DMEM and DMSO control groups. (C) Population 1 was treated with 200 μM, 100 μM and 50 μM of RG108 for 7 days; only 50 μM showed similar metabolic activity to DMEM group. (D) Population 1 was treated with 50 μM RG108 for 3 days confirming this concentration has little effect on hBMSCs viability. Three biological replicates were used for each experiment and each one was plated in six technical repeats and the cells’ metabolic activity was measured using the 3-(4,5-dimethylthiazol-2-yl)-2,5-diphenyltetrazolium bromide (MTT) assay. The data are presented relative to DMEM group as mean and standard deviation. For all graphics, asterisks above the bars represent significant inter-group differences when compared to DMEM. Other significant differences are represented by asterisks above the linkers. *, ** and *** indicate, respectively, p ≤ 0.01, p ≤ 0.001 p ≤ 0.0001 by ANOVA One Way followed by the Tukey test (c and d) and by Kruskal-Wallis followed by the Dunn's Multiple Comparison Test (a and b).

The optimal RG108 treatment in hBMSCs would result in DNA demethylation and enhance the cells’ multipotency without any toxic effects. Therefore, we first tested different inhibitor concentrations for their effects on cell viability, apoptosis and population doubling times. In the first set of experiments, two hBMSCs populations were treated for 7 days with high concentrations (400 μM, 800 μM and 1.6 mM) of the compound. Significant decrease in cell viability was observed in all of them when compared to DMEM and 1.6 mM compared to DMEM and DMSO controls (Fig 2A and 2B). Subsequently 50 μM, 100 μM, 200 μM RG108 treatments were tested at day 7 in one of the hBMSCs populations (Fig 2C). Metabolic activity was not affected when the cells were treated with 50 μM of the compound. After choosing 50 μM as optimal concentration, the hBMSCs’ viability was again evaluated at day 3. The observed decrease in cell’s viability is about 5% in the RG108 and DMSO-treated groups comparing to DMEM therefore the effect was small even if significant (Fig 2D).

**Fig 3:**
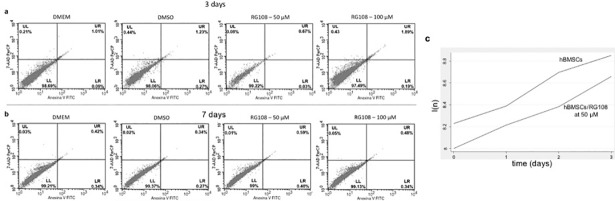
Effect of RG108 on apoptosis in hBMSCs. (A, B) hBMSCs were treated for 3 and 7 days with 50 μM and 100 μM RG108 and compared to DMEM and DMSO controls. Neither of the treatments triggered cell death by apoptosis at 3 and 7 days. The non-apoptotic cells are sorted into the lower left (LL) quadrant. (C) Three days of 50 μM RG108 treatment did not affect also the hBMSCs’ ability to proliferate in vitro. Two biological replicates were performed for each group; data are presented as l(n) of cells seeded at three different densities and quantified spectrophotometrically at 260 nm.

In addition, an apoptosis assay was performed at Day 3 and 7, for 50 μM and 100 μM of RG108. Again, no differences were observed, with DMEM group having 98.69% of intact cells and DMSO and RG108 (50 μM and 100 μM) groups having 98.06%, 99.22% and 97.49%, respectively (Fig 3A) at 3 days; similar results were obtained at 7 days (Fig 3B).

The population doubling time (PDT) of RG108/50 μM-treated cells did not differ from the control cells (43.73h and 43.23h, respectively), demonstrating that 3 days of 50 μM RG108 treatment does not affect the ability of hBMSCs to proliferate *in vitro* (Fig 3C).

### Effect of RG108 on global DNA methylation and hydroxymethylation levels

**Fig 4:**
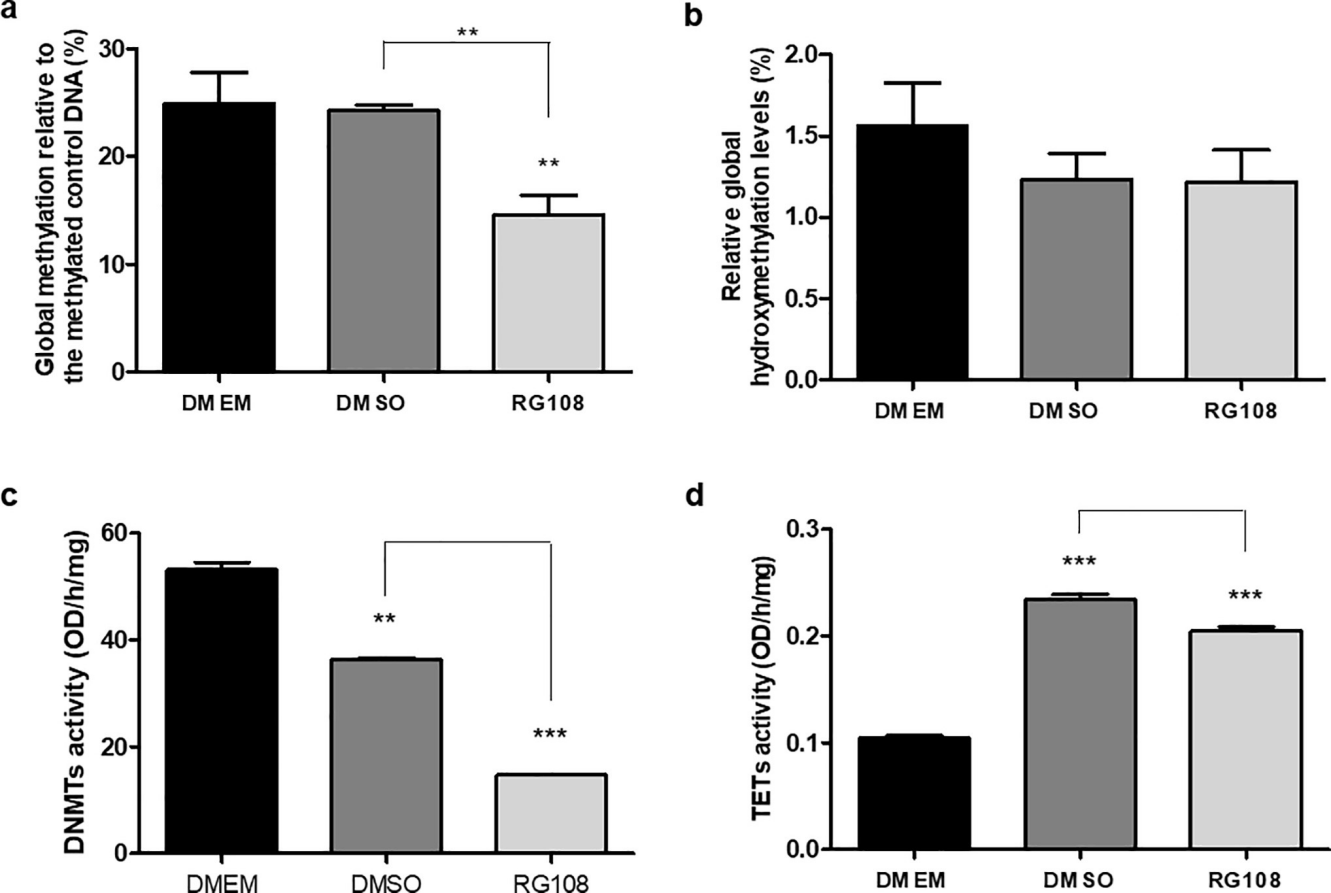
Global effects of RG108 on DNA modifications and DNMTs and TETs enzymatic activities in hBMSCs. hBMSCs were treated with 50 μM RG108 for 3 days and compared to DMEM and DMSO controls. (A) Global methylation was assessed using Imprint Methylated DNA Quantification Kit showing a significant decrease in the RG108 group. (B) Global hydroxymethylation was analyzed using Quest 5-hmC DNA Elisa kit; no statistical differences are observed amongst groups. DNMTs (C) and TETs (D) enzymatic activities were assessed using colorimetric assays. Global methylation and hydroxymethylation experiments were performed in biological triplicates and DNMTs and TETs activities were performed in biological duplicates. For all graphics, ** or *** above the bars represent significant inter-group differences when compared to DMEM. Other significant differences are represented by * symbol above the linkers. ** and *** indicate, respectively, p ≤ 0.001 and p ≤ 0.0001 by ANOVA One Way followed by the Tukey test.

The global DNA methylation was assessed by a colorimetric assay. After samples were compared to the methylated control sample, the methylation levels were observed to be significantly lower in cells treated with 50 μM of RG108 for 3 days when compared to DMEM and DMEM/DMSO (p ≤ 0.001) groups. Addition of DMSO alone did not affect the methylation levels (Fig 4A). Next, a colorimetric assay was used to analyse global DNA hydroxymethylation levels. After samples were compared to the hydroxymethylated control sample, both RG108 and DMSO treated cells showed lower levels of global hydroxymethylation comparing to the DMEM group, however, these differences were not statistically significant (Fig 4B).

### RG108 reduces the DNMTs’ activity while DMSO is responsible for an increase in the TETs’ activity

Since the RG108 compound was specifically designed to block the DNMTs active site and a decrease in the global methylation levels was observed, it was expected to see a decrease in the DNMTs’ activity in nuclear protein extracts. Indeed, there was a significant (63%) reduction in the DNMTs activity levels for 50 μM RG108 when compared to both DMEM (p ≤ 0.0001) and DMSO-treated cells (p ≤ 0.05). A small although significant decrease in DNMTs’ activity was also observed for DMSO-treated cells (Fig 4C); however, this was not sufficient to affect global DNA methylation levels (Fig 4A). The activity levels of demethylases (TETs) were also evaluated and a significant increase was observed, to a similar extent, comparing to DMEM, for both DMSO and RG108 treated cells (Fig 4D). However, the effect was bigger for DMSO, comparing to RG108 (p ≤ 0.01).

### RG108 changes gene expression of epigenetic machinery enzymes and multipotency markers

**Fig 5:**
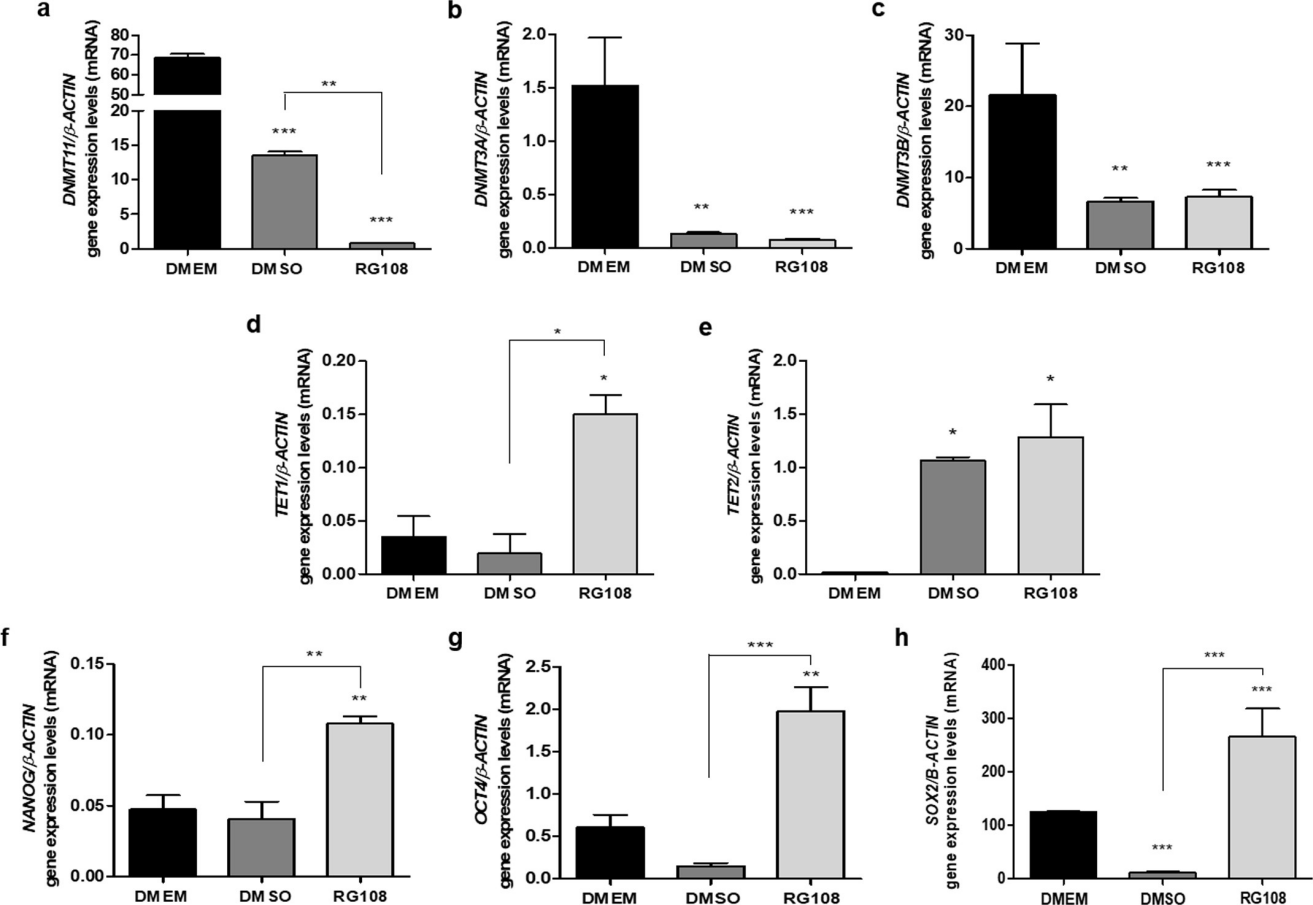
Changes in transcript levels of epigenetic machinery genes and multipotency markers after RG108 treatment. *DNMT1* (A), *DNMT3A* (B), *DNMT3B* (C), *TET1* (D), *TET2* (E), *NANOG* (F), *OCT4* (G) and *SOX2* (H) mRNA levels were analyzed by qPCR after 3 days of treatment with 50 μM of RG108. Results correspond to mRNA levels ratio normalized to the β-actin levels. Determination of gene expression relative levels was performed using the cycle threshold (Ct) method and the results are presented as mean/SD. Two biological replicates were performed for each group and each of the biological replicates was analyzed in technical triplicates. For all graphics, *, ** or *** above bars represent significant inter-group differences when compared to DMEM. Other significant differences are represented by * symbol above the linkers. *, ** and *** indicate, respectively, p ≤ 0.01, p ≤ 0.001 p ≤ 0.0001 by ANOVA One Way followed by the Tukey test.

Although the decrease in DNMTs’ activity after RG108 treatment can be explained by blocking the DNMT1 active site by the compound, we further investigated whether *DNMT1*, *DNMT3A* and *DNMT3B* genes transcripts were also affected. Results demonstrated a large, 50%-95%, decrease in *DNMTs* transcript levels in RG108 (*DNMT1)* and DMSO-treated cells (*DNMT3A* and *DNMT3B*) compared to DMEM control which could contribute to the substantial loss of methylation observed in hBMSCs. For the maintenance *DNMT1*, the most highly expressed DNA methyltransferase in these cells, the decrease was also significant when compared to the DMSO control samples (p ≤ 0.001) (Fig 5A–5C).

In addition to the effect on *DNMT1*, RG108 treatment appears to upregulate the expression of TETs, mostly *TET1*. A significant increase in *TET1* mRNA level was observed in RG108-treated cells when compared to both DMSO (p ≤ 0.05) and DMEM (p ≤ 0.001) groups (Fig 5D). Although DMSO did not modulate *TET1* transcripts, the DMSO-promoted increase in the *TET2* gene expression was statistically significant, comparing to DMEM (p ≤ 0.001) (Fig 5E). We did not detect any PCR product for *TET3* and it is possible this enzyme is not expressed or very low expressed in hBMSCs.

mRNA levels of *SOX2* (RG108 X DMSO p ≤ 0.0001; RG108 X DMEM p ≤ 0.0001), *NANOG* (RG108 X DMSO p ≤ 0.001; RG108 X DMEM p ≤ 0.001) and *OCT4* (RG108 X DMSO p ≤ 0.0001; RG108 X DMEM p ≤ 0.001) were significantly increased in RG108-treated cells and this effect was independent of DMSO (Fig 5F–5H).

### Gene-specific changes in methylation and hydroxymethylation after RG108 treatment

Methylation and hydroxymethylation changes at *DNMT1*, *DNMT3A*, *DNMT3B*, *TET1*, *TET2*, *TET3*, *NANOG* and *OCT4* genes were evaluated by quantitative-methylation-PCR assay method. The analysed regions are within the promoters (+/-1.5kB from the transcription start site) and are characterized by H3K4Me1, H3K4Me3 and H3K27Ac marks, DNA I hypersensitivity clusters and regulatory elements as well as increased density of CpG sites.

**Fig 6:**
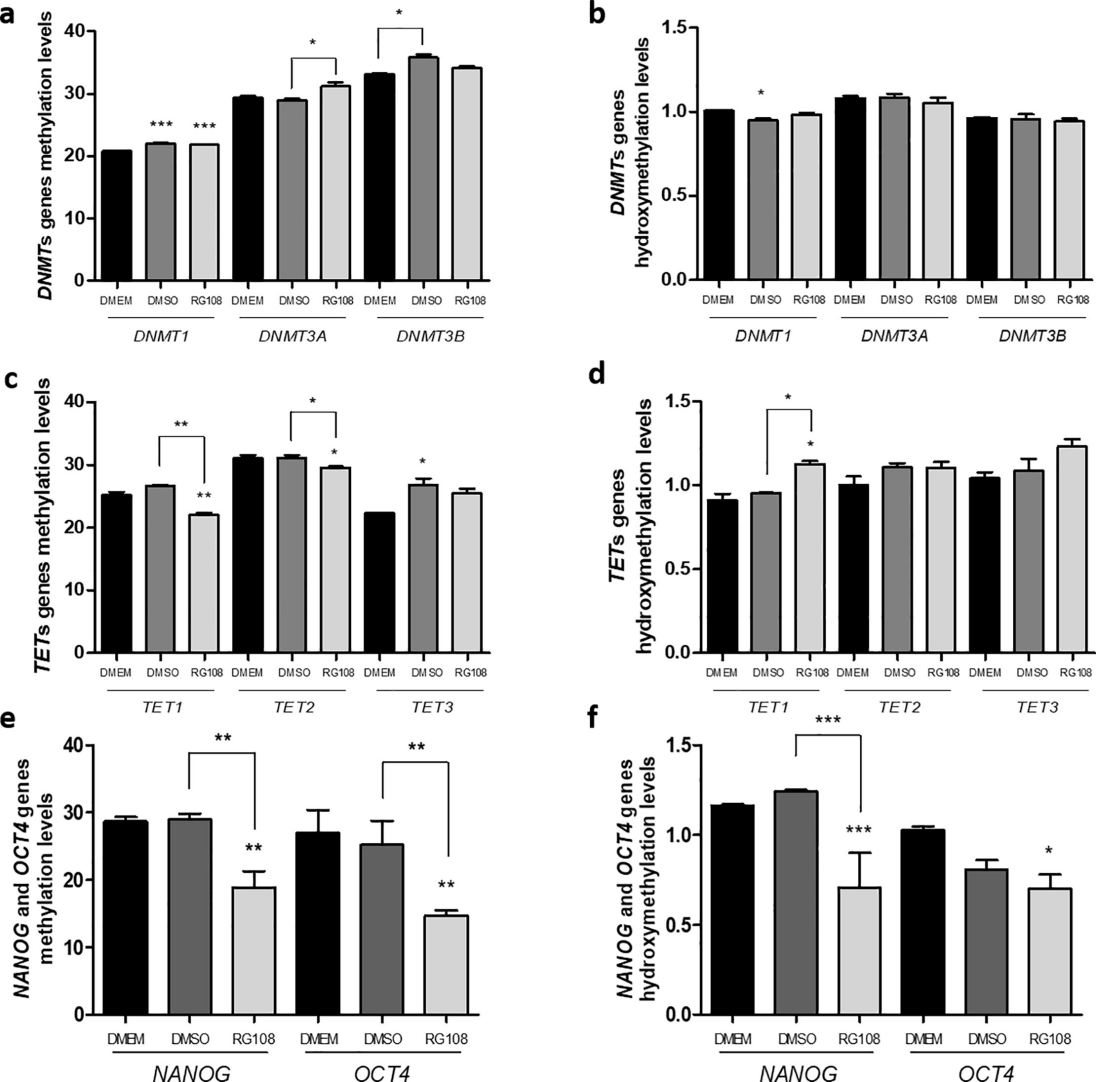
Changes in DNA modifications at gene-specific regulatory elements in response to RG108 treatment. Methylation and hydroxymethylation levels were analyzed by DNA glycosylation followed by restriction enzyme analysis and qPCR of promoter sequences, after 3 days of 50 μM RG108 treatment and compared to DMEM and DMSO controls. The relative levels were determined using the cycle threshold (Ct) method and the methylation results are presented as *HpaII* levels—*MspI* levels/control levels and the hydroxymethylation results are presented as *MspI* levels/control levels (% of control). Two biological replicates were performed for each group and each of the biological replicates was done in technical triplicates. (A) All *DNMT* genes are mostly unmethylated (20–30% methylation level) and undergo only small (2–3%) methylation after RG108. (B) The hydroxymethylation levels at DNMTs’ promoters are low and do not change after RG108 treatment. (C) *TET* genes have similar 20–30% methylation levels; *TET1* and *TET2* undergo small but significant demethylation after RG108. (D) Hydroxymethylation levels at TET genes are low and do not change with RG108. (E) RG108 treatment resulted in 40% and 40% loss of methylation and hydroxymethylation, respectively at NANOG regulatory element. (F) For OCT4, the methylation loss was 48% and hydroxymethylation was 32%. For all graphics, *, ** or *** above the bars represent significant inter-group differences when compared to DMEM. Other significant differences are represented by * symbol above the linkers. *, ** and *** indicate, respectively, p ≤ 0.01, p ≤ 0.001 p ≤ 0.0001 by ANOVA One Way followed by the Tukey test.

All analysed regulatory regions for DNMTs were in mostly unmethylated state (20–30% methylation) therefore permissive of transcription (Fig 6A). Any RG108 or/and DMSO-triggered increases in methylation were very small (2–3%) and unlikely to account for the substantial changes in gene expression (Fig 5). Similarly, there was no change in hydroxymethylation levels at these genes.

On the other hand, both *TET1* and *TET2* (but not *TET3*) genes have demonstrated a statistically significant decrease in the methylation levels for the RG108 group, comparing to DMEM (p ≤ 0.01) (Fig 6C) which corresponds with the increase in their transcript levels (Fig 5). An additional modulation in the hydroxymethylation levels in the *TET1* gene (p ≤ 0.01) was observed in the RG108 group, comparing to DMEM and DMSO groups (Fig 6D).

Bigger changes after RG108 treatment were observed at the multipotency genes. The *NANOG* gene demonstrated a statistically significant 40% loss of methylation comparing to DMSO (p ≤ 0.01) and to DMEM (p ≤ 0.01) (Fig 6E). The hydroxymethylation levels were also significantly lower for the RG108 group (Fig 6F) (p ≤ 0.01 comparing to DMEM and DMSO). Furthermore, the methylation and hydroxymethylation levels at the *OCT4* gene region were significantly reduced (almost 50% loss of methylation) for the RG108 group, when compared to the DMEM group (p ≤ 0.01) (Fig 6E) and the hydroxymethylation loss was also significantly different between the RG108 and DMEM group (p ≤ 0.01) (Fig 6F).

### RG108 increases the mesenchymal stem cell markers NANOG, OCT4 and CD105

**Fig 7:**
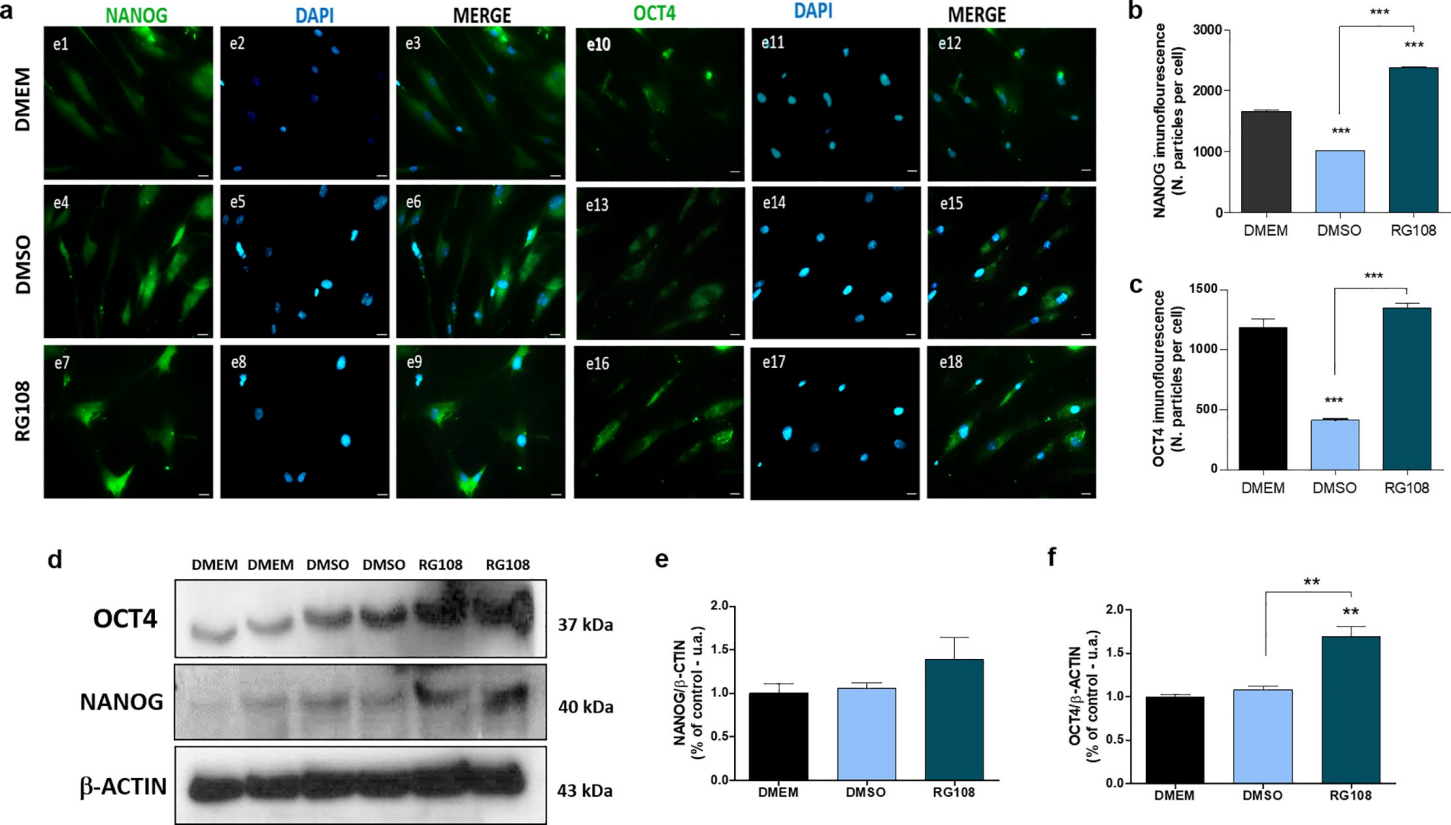
RG108-trigerred changes to multipotency gene markers. hBMSCs were treated with 50 μM of RG108 for 3 days and subject to immunostaining (A, B and C) and western blot (D, E and F) analysis. Untreated (DMEM) and vehicle control-treated (DMSO) cells were used as controls. Two biological replicates were performed for each group. The data show a significant increase in the immunostaining of NANOG (A and B) and OCT4 (A, C, D and F). Representative images are shown in A and D. Nuclei were labelled with DAPI (blue). Magnification x40. For all graphics, ** over the bars represent significant inter-group differences when compared to DMEM. Other significant differences are represented by * symbol above the linkers. ** and *** indicate, respectively, p ≤ 0.001 p ≤ 0.0001 by ANOVA One Way followed by the Tukey test.

In line with the gene expression results, an increase in the OCT4 and NANOG immunostaining was observed in the RG108-treated cells (Fig 7A, 7B and 7C), as well as in the OCT4 and NANOG protein content, with statistical difference for OCT4 (RG108 X DMSO p ≤ 0.001; RG108 X DMEM p ≤ 0.001) (Fig 7D, 7E and 7F). These data corresponded to the increased gene expression observed for *NANOG* and *OCT4* (Fig 5F and 5G).

**Fig 8:**
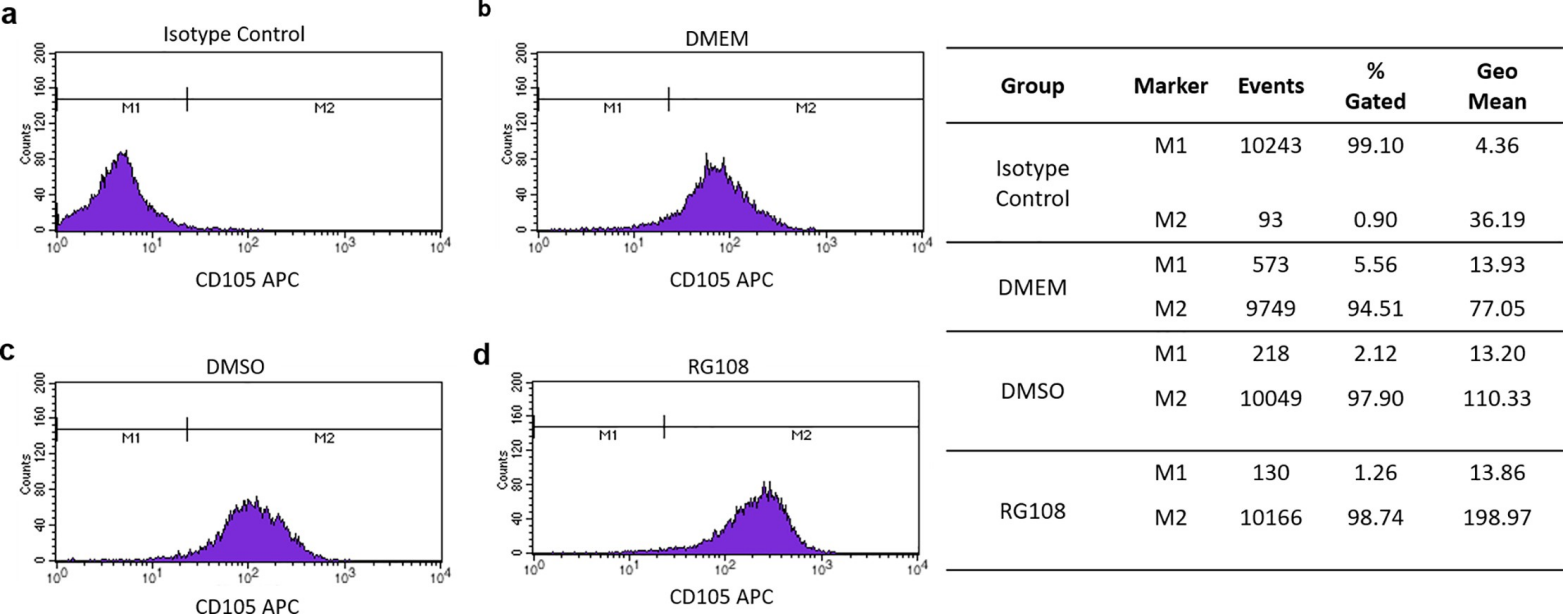
Effect of RG108 on CD105 surface marker. Flow cytometry histograms of hBMSCs showed modulation of CD105 surface marker, after RG108 and DMSO treatments, for three days. hBMSCs were labelled with allophycocyanin (APC)-conjugated monoclonal antibody; positive cells are located at M2 region and M1 represents isotype as control.

The mesenchymal cell surface marker CD105 was increased after DMSO treatment (~1.5 times) (Fig 8C); however, the RG108 treatment promoted bigger changes, increasing the CD105 positive signal from 77.05 (DMEM group, Fig 8B) to 198.97 (RG108 group—~2.5 times more, Fig 8D).

## Discussion

Improvement of cell therapy efficacy is one of the main goals in regenerative medicine and hMSCs have been extensively studied for this purpose. However, their epigenetic regulation remains to be elucidated. Epigenetic mechanisms contribute significantly to phenotype changes and cell fate specification. Therefore, understanding the hMSCs epigenetics is crucial for planning and executing stem cell-based therapies. This study aimed to test whether the DNMT1 inhibitor, RG108, can be used to increase mesenchymal cell markers in hBMSCs and to investigate the underlying epigenetic mechanisms. The results show that treatment of hBMSCs with 50 uM RG108: (i) has no cytotoxic effects; (ii) leads to significant loss of global DNA methylation; (iii) results in significant increase in RNA and protein levels of NANOG and OCT4 that correlates with a local decrease in DNA methylation at their regulatory elements; and (iiii) increases the mesenchymal cell surface marker CD105.

RG108 is a non-nucleoside inhibitor that blocks the DNMT1 active site and shows fewer side effects than other inhibitors [10]. Importantly, recent investigations have suggested that RG108 may provide increased hMSCs efficacy in stem cell therapy [19,20]. In the present study RG108, when used at 50 μM and 100 μM, showed lack of toxicity and did not trigger cell death by apoptosis or changes in the cells’ ability to proliferate *in vitro*. These results are in line with previous observations reported for hBMSCs [19,20]. Such observations might be cell type-specific and dependent on epigenetic background as in human prostate cancer cells similar concentrations of RG108 led to growth inhibition and apoptosis [14].

hBMSCs have never been subjected to global epigenomic analysis after RG108 treatment. As expected, our results showed a substantial (75%) decrease in the DNMTs activity and this was followed by a reduction in global DNA methylation levels. The observed decrease was approximately 42% in RG108 group and this result is much higher than 10% reported by Graça et al. in prostate cancer cell lines [14]. Although RG108 works primarily through blocking DNMT1 catalytic site we also observe a large decrease in *DNMT1* transcripts which could contribute to the extensive demethylation observed. Interestingly, this downregulation was also reported in prostate cancer cells after RG108 treatment [14]. The mechanism of DNMTs suppression is not known and the observed small increase (~2%) in promoter methylation is unlikely to be responsible for it. DNMTs contribute to formation of higher-order chromatin structure leading to gene silencing [26–28]. The iPS reprogramming process requires an open chromatin state [29] and DNMTs might suppress a decondensation process of the chromatin and therefore prevent the accessibility of the reprogramming factors to their target regions. RG108 has been previously used in iPS reprogramming and it is possible its effects on DNMTs and chromatin structure are similar to these observed in hBMSCs.

It is also probable that RG108 could affect, through secondary mechanisms, the DNA hydroxymethylation levels and demethylases activity, but this has never been studied. Here we show that *TET1* expression was upregulated which suggests that the hydroxymethylation levels could increase if longer treatment was applied. This could additionally contribute to the decrease in DNA methylation. The upregulation of *TET1* and *TET2* genes correlated with a significant decrease in local methylation and, for *TET1*, increase in hydroxymethylation levels.

Most importantly, RG108 treatment in hBMSCs leads to a significant upregulation of two multipotency genes, *NANOG* and *OCT4*, which was observed at mRNA level by gene expression analysis and protein, by immunostaining and western blot analysis. The upregulation of *NANOG* and *OCT4* correlates with RG108-trigerred decrease in methylation and hydroxymethylation at their regulatory elements. Some studies have shown that TET1 transcript levels are regulated by OCT4, a multipotency marker and it can play an important role in DNA hydroxymethylation and methylation during cells reprogramming [30]. To be considered mesenchymal stem cells, cells must express specific surface markers, such as CD105 [31]. Also known as Endoglin, CD105 is a transmembrane receptor for TGF-ß family and it is expressed in hBMSCs [32]; its activation is associated with cell proliferation and osteogenic differentiation [33] through TGF-ß signaling pathway (smad-dependent/smad-independent and/or MAPK signaling). In addition to the upregulation of NANOG and OCT4, RG108-treated cells showed upregulation of CD105 (~2.5 times), a well-known MSCs surface marker. This increase was also observed in mesenchymal cells derived from human periodontal ligament (hPDLSCs) (data not shown), although we did not observe the increase in the NANOG and OCT4 at mRNA and protein levels in hPDLSCs (data not shown); therefore, the RG108 effect might be cell specific. In addition, the compound was not tested during cell differentiation processes, such as adipogenic or osteogenic induction.

Previously, TET1 and NANOG proteins have been shown to co-occupy genomic loci of genes associated with the maintenance of pluripotency and lineage commitment [5]. Furthermore, NANOG recruit TET1 to enhance the expression of multipotency markers such as OCT4 [5]. Subsequently Olariu et al., proposed a model in which the regulatory network of OCT4, NANOG and TET1 includes positive feedback loops involving DNA-demethylation around *OCT4* and *TET1* promoters [34]. Although the present investigation was performed in hBMSCs, OCT4, NANOG and TET1 play similar roles to those in embryonic stem cells and are essential for epigenetic regulation of cell multipotentiality. Epigenetics changes such as DNA hydroxymethylation promoted by TET1 are important to modulate regulatory regions of multipotency markers [30] and *TET1* is capable of modulating DNA methylation levels at CpG-rich promoters of multipotency markers, as *OCT4* [30]. Our results show that RG108 treatment could in fact affect this complex.

Based on the obtained results and published data we propose a mechanism of RG108 action in hBMSCs which originates from an inhibition of the enzymatic activity of DNMTs, leading to a global decrease in DNA methylation and a local decrease of methylation levels at the promoters of *OCT4* and *NANOG* which, in turn, would upregulate their RNA and protein content. In addition, downregulation in DNMT1 expression and an increase in TET1 expression and activity would further enhance global loss of methylation. The global loss of methylation, high levels of OCT4/NANOG/TET1 and changes in their epigenetic patterns could then promote a more permissive chromatin therefore reinforcing the state of multipotency in hBMSCs, since OCT4/NANOG/TET1 are well known to promote cell multipotency.

Due to rigorous use of vehicle (DMSO) control we could observe the DMSO effects on epigenetic regulation and dissociate them from the effects of RG108. DMSO was responsible for the change in TETs activity and contributed to the modulation of DNMT3A, DNMT3B and TET2 gene expression. DMSO has been previously reported to affect epigenetic regulation, possibly through interactions with DNA and substrates like AdoMet [35,36]. Therefore, taking previously published reports and results obtained in this study the DMSO might act as a modulator of the epigenetic machinery genes and this possibility should be taken into account when using DMSO as a vehicle. However, it should be underlined that in the present study only RG108 but not DMSO had a significant impact on multipotency genes expression and their DNA methylation/hydroxymethylation levels. Therefore, the suggested supportive role of RG108 in hBMSCs-based stem cell therapies is due to the drug itself.

## Conclusions

When used at 50 μM, RG108 is a non-cytotoxic demethylating agent, triggering changes in DNA methylation/hydroxymethylation levels, enzymatic activity of epigenetic machinery and increases mesenchymal cell markers such as NANOG, OCT4 and CD105, in hBMSCs. The epigenetic modulation might be involved in the chromatin remodelling, leading to a more permissive chromatin state and these changes could contribute to the proposed role of RG108 in improving the efficacy of stem cell therapies. DMSO also triggered changes, suggesting that DMSO can be considered a modulator of epigenetic machinery enzymes, although with milder effect compared to RG108.
